# Evaluation of a strategy to shorten the time to surgery in patients on antiplatelet therapy with a proximal femur fracture (AFFEcT Study): Study protocol for a multicenter randomized controlled clinical trial

**DOI:** 10.1097/MD.0000000000015514

**Published:** 2019-05-13

**Authors:** Anaya Rafael, Rodriguez Mireia, Gil José María, Moral Victoria, Millan Angélica, Vilalta Noèlia, Delgado Claudia Erica, Antonijoan Rosa María, Reguant Francesca, Guilabert Patricia, Blanco Domingo, Mateo José, Merchán-Galvis Angela, Martinez-Zapata Maria Jose

**Affiliations:** a Anesthesiology Service, Hospital de la Santa Creu i Sant Pau; b Orthopedic Surgery and Tramatology Service, Hospital de la Santa Creu i Sant Pau; c Hematology Service, Hospital de la Santa Creu i Sant Pau; d Clinical Pharmacology Service, Fundació Institut de Recerca Hospital de la Santa Creu i Sant Pau; e Clinical Pharmacology Service, Hospital de la Santa Creu i Sant Pau; f Anesthesiology Service, Xarxa Assitencial Universitària de Manresa; g Anesthesiology Service, Hospital de la Vall d’Hebron; h Public Health and Clinical Epidemiology Service-Iberoamerican Cochrane Centre, IIB Sant Pau, Spain; i Departamento de Medicina Social y Salud Familiar, Universidad del Cauca, Colombia; j CIBERESP, Spain.

**Keywords:** randomized clinical trial, platelet aggregation inhibitors, femoral fracture, platelet function

## Abstract

**Introduction:**

Patients with femur fracture benefit from early surgery. Recent reports suggest that regional anesthesia may be superior to general anesthesia in these patients. Early surgery under spinal anesthesia could be performed safely by determining platelet function in patients receiving antiplatelet agents.

**Methods:**

Multicenter, randomized, open-label, parallel clinical trial expected to include 156 patients ≥ 18 years of age under chronic treatment with antiplatelet agents who develop a proximal femur fracture. Exclusion criteria: presence of multiple or pathological fractures, current treatment with vitamin K antagonists or new oral anticoagulants, and congenital or acquired coagulopathy.

Patients will be randomized to either

1. a guided early surgery strategy in which preoperative platelet function will be assessed at emergency department admission or
2. a conventional strategy in which surgery under neuraxial anesthesia will be scheduled according to the margin of safety time established for the specific antiplatelet agent.

The primary endpoint is time (hours) from admission to surgery. Secondary endpoints include: platelet function; postoperative bleeding; medical-surgical complications; perioperative and 1-year mortality; quality of life; length of hospital stay; cost-effectiveness; and cost-utility. Follow-up assessments will be performed during hospital admission and at 1, 6, and 12 months after surgery.

**Potential impact of the study:**

The determination of platelet function at admission to the emergency department in patients with femoral fracture receiving antiplatelet therapy may permit earlier surgery under spinal anesthesia, thus shortening the hospital stay and reducing the risk of complications. These advantages associated with early surgery could positively impact patient well-being and also reduce treatment-related healthcare costs.

**Ethics and dissemination:**

The study has been approved by the ethics committees at all participating centers. Their results will be disseminated in congresses and published in peer reviewed journals.

## 1 Introduction

Femoral fractures in elderly patients are the most common cause of admission to Orthopaedic Surgery and Traumatology departments. Proximal femoral fractures account for approximately 42–50% of all fractures in the elderly population.^[1]^ Postoperative mortality rates in the first 30 days can be as high as 10% and range from 18–33% in the first year. Several patient-related variables—including older age, males, dementia, and frailty—are associated with higher mortality rates.^[2–6]^ The annual cost to the health care system of treating femoral fractures is non-negligible, primarily due to high morbidity and mortality rates.^[7,8]^

Clinical guidelines recommend that surgery be performed, if feasible, within the first 48 hours after fracture in order to reduce perioperative complications and in-hospital morbidity and mortality.^[9,10]^ Although studies have shown that early surgery is beneficial for patients on chronic antiplatelet therapy,^[11]^ these patients must undergo general anesthesia because neuraxial anesthesia is contraindicated.^[12–15]^ The choice of anesthetic technique in patients with femoral fracture is controversial due to influence of the anesthetic technique on postoperative morbidity and mortality. Based on recent data, neuraxial anesthesia yields lower in-hospital mortality rates and shorter hospital stays than general anesthesia in patients with femoral fracture.^[16,17]^ However, more evidence is needed to definitively resolve this debate. In this regard, 2 clinical trials are currently underway to compare these 2 anesthetic techniques.^[18,19]^

Some clinical guidelines recommend platelet function testing to shorten the time from admission to surgery in patients who undergo neuraxial anesthesia.^[20,21]^ In patients receiving chronic antiplatelet treatment, the use of these tests to determine the number of functional platelets is crucial to ensure that early surgery with neuraxial anesthesia can be performed safely.

In this context, we have designed a randomized clinical trial to evaluate the safety and efficiency of a therapeutic strategy involving preoperative determination of platelet function in patients with femoral fracture receiving chronic antiplatelet treatment. The main objective of this trial is determined whether this strategy, designed to reduce the time from admission to surgery under spinal anesthesia, provides any benefits compared to a conventional strategy based on delayed surgery after discontinuation of antiplatelet therapy.

## 2 Methods

The study protocol (version 3, code IIBSP-PLA-2016–86) was developed in accordance with the SPIRIT recommendations for interventional trials.^[22]^ The present randomized controlled trial was approved by the ethics committees of all participating centers and registered at clinical trials.gov (NCT03231787).

### 2.1 Study design

Multicenter, randomized, open-label, parallel clinical trial of patients receiving chronic antiplatelet therapy admitted to the emergency department with a femoral fracture. All study participants will undergo platelet function testing and those with more than 80,000 functional platelets will be randomized to either early surgery with neuraxial anesthesia or delayed surgery with neuraxial anesthesia performed in accordance with the antiplatelet activity time of the specific antiplatelet drug (3 days for aspirin 300 mg/day and triflusal 600 mg/day; 5 days for clopidogrel and ticagrelor; 7 days for prasugrel, and 10 days for ticlopidine).

Recruitment started in September 2017 and will continue until December 2019. The last follow-up visit for the final patient included in the study will be in December 2020. To date, 81 patients have been included.

### 2.2 Objectives

#### 2.2.1 Primary

To compare the time in days from hospital admission until surgery in the 2 groups.

#### 2.2.2 Secondary

To check for difference between the 2 groups in the following variables: postoperative bleeding, medical and surgical complications, length of hospital stay, quality of life, mortality, cost-effectiveness, and cost-utility.

Agreement between the 2 different platelet function assays (see Methods) will be assessed in a subset of 20 patients.

### 2.3 Participants

#### 2.3.1 Study location

The following 4 centers will participate in this study: Hospital de la Santa Creu and Sant Pau, Althaia-Xarxa Assistencial Universitària de Manresa,Vall d’Hebron Hospital, and Hospital Clinic of Barcelona (Spain).

#### 2.3.2 Inclusion criteria

Study inclusion criteria:

(1) adults (male or female) ≥ 18 years of age;
(2) diagnosis of femoral fracture; and
(3) receiving treatment with antiplatelet agents (acetylsalicylic acid [ASA] > 100 mg/d; triflusal > 300 mg/d; clopidogrel; prasugrel; ticagrelor; or ticlopidine) at admission to the emergency department.

Written informed consent will be obtained from all patients.

#### 2.3.3 Exclusion criteria

Exclusion criteria: presence of multiple or pathological fractures, current treatment with vitamin K antagonists or new oral anticoagulants, and congenital or acquired coagulopathy.

#### 2.3.4 Criteria for withdrawal from the clinical trial

The emergence of any of the following events may justify study withdrawal: severe adverse event, any clinical condition preventing the patient from continuing in the trial, protocol violations, the patient's voluntary decision to withdraw, loss to follow-up, or death.

### 2.4 Outcomes and measures

#### 2.4.1 Main outcome

Time, in hours, from emergency room admission until surgery.

#### 2.4.2 Secondary outcomes

Clinical: postoperative bleeding, complications associated with the surgical procedure and surgical wound, medical complications during admission, perioperative mortality and mortality at 1 year, quality of life (EQ-5D-5L generic questionnaire) assessed preoperatively and postoperatively at days 5 and 30 and months 6 and 12.

Costs: length of hospital stay, surgery-associated direct health costs, laboratory tests, medications, consumables, complementary diagnostic tests, resources required to treat perioperative complications, cost associated with rehabilitation, consultations with specialists, and primary care visits after hospital discharge and during follow-up.

A subgroup analysis of 20 consecutive patients from one of the participating centers will be performed to determine the correlation between 2 platelet function systems (PlaTeLet work [PTLwork] versus the conventional Platelet Function Assay [PFA-100 system]).

#### 2.4.3 Patient follow-up

Follow-up visits will be conducted during hospitalization, and postoperatively by phone at months 1, 6, and 12.

### 2.5 Randomization and allocation of interventions

A computer-generated permuted block randomization sequence, stratified by center, will be used for the randomization process. A web-based platform (www.clipnasis.com) will be used to allocate patients to the interventions. This platform will randomly assign a number to each patient and to the corresponding intervention. Researchers will be blinded to the allocation sequence of the study interventions.

### 2.6 Blinding

Necessarily, the patients and researchers will know which intervention they have been allocated. However, to avoid influencing the timing of the surgical procedure, the investigators will be blinded to the platelet function status in the control group.

### 2.7 Implementation and data collection

**Figure 1:**
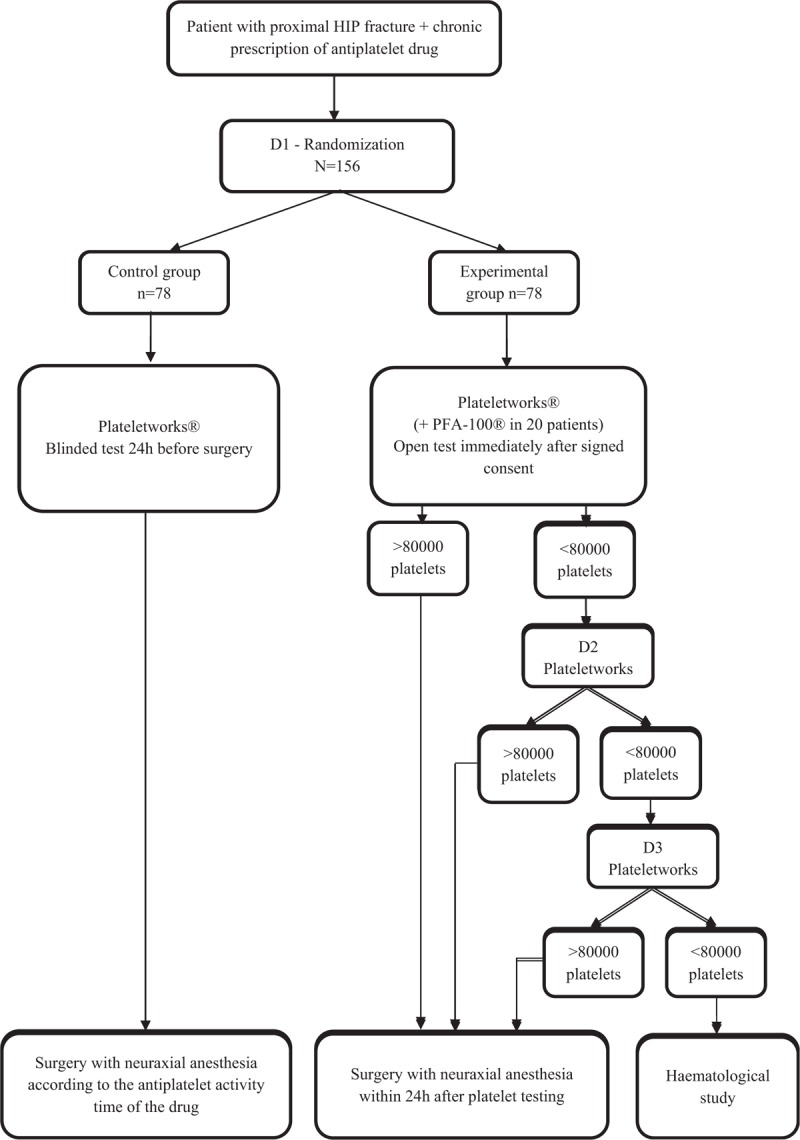
Flowchart of the clinical trial.

Figure 1 describes the patient recruitment process and the study plan. A competitive recruitment process will be used; thus, no predetermined limit to the total number of patients per center has been established. Patient selection will be performed on the day of admission to the hospital emergency department. Researchers will first verify that the patient meets the study inclusion criteria. The patient will then be informed about the clinical trial and asked to participate. Patients who agree to participate will be enrolled in the study after signing the informed consent form. Next, they will be randomized to one of the 2 study groups.

Blood samples will be taken at the time points established in the study and analyzed in the laboratory within 10 minutes of extraction to determine platelet function. Platelet function will be performed in all patients using the PFA-100 system (Siemens, Munich, Germany). This system is based on the adhesion properties of platelets when subjected to stress conditions and in the presence of a particular agonist. The citrated whole blood is conducted through a capillary to a collagen membrane with a microscopic opening (147 μm) covered by an agonist (adenosine diphosphate [ADP] or epinephrine). The system determines the time it takes for the hole to close (closure time).

In a small subset of patients (n = 20), the PTLwork system (Helena Laboratories, Beaumont, TX) will also be used to determine preoperative platelet function. This 2-step system determines the number of platelets added and/or inhibited. In the first step, the total platelets are counted in a blood sample. Next, a second sample containing a platelet agonist (ADP or collagen) is examined to count the number of non-functional platelets. The difference between the 2 platelet counts yields the number of functioning platelets.^[23]^

The key outcome variables of this clinical trial (date and time of admission; date and time of surgery; blood loss; transfusion units; and complications) will be obtained from patient medical records and by direct patient interview (in person and/or by telephone).

The collected information will be uploaded to a password-protected electronic web-based database (http://www.clinapsis.com).

### 2.8 Interventions

#### 2.8.1 Experimental intervention

At emergency room admission, the antiplatelet medication will be discontinued and platelet function will be determined. In patients with >80,000 functional platelets, the surgery will be scheduled within the next 24 hours. However, if the level of functional platelets is <80,000, then the platelet assay will be performed once per day for the next 2 days or until normalized (whichever occurs first): surgery will be performed within 24 hours after normalization. If the number of functional platelets has not normalized after 3 days, surgery with neuraxial anesthesia will be scheduled in accordance to the margin of safety time established for the specific antiplatelet medication.

#### 2.8.2 Control intervention

Antiplatelet therapy will be discontinued at admission to the emergency room. Surgery with neuraxial anesthesia will be performed in accordance with the established safety time for the antiplatelet drug. Platelet function will be determined the day before surgery but the results will not be revealed to the researchers until the end of the study.

#### 2.8.3 Co-intervention

All included patients must be eligible for neuraxial anesthesia.

#### 2.8.4 Description of the surgical intervention

The specific surgical technique—osteosynthesis, hemiarthroplasty, or total hip arthroplasty—will depend on the characteristics of the proximal femur fracture in each patient. Osteosynthesis will be performed with cannulated screws, Dynamic Hip Screw with plate (DHS), or intramedullary nail (short or long), depending on the fracture type. Displaced subcapital fractures will be treated by hemiarthroplasty (monopolar or bipolar) or total hip arthroplasty, depending on the patient characteristics. Total arthroplasty can be cemented, uncemented, or hybrid. In all cases, the orthopedic surgeon will follow the surgical protocol in place at the treating hospital for the type of fracture. Each center will follow the specific transfusion protocol in place at that institution.

### 2.9 Statistics

#### 2.9.1 Sample size estimation

One of the participating centers used historical data from the hospital database to calculate the mean time from admission to surgical intervention in patients not receiving antiplatelet treatment and without any coagulopathies: 2.85 days (standard deviation [SD], 3.17). Accepting an alpha risk of 0.05 and a beta risk under 0.20 in a 2-sided contrast and a 10% loss rate, the estimated sample size needed to detect a decrease of ≥1.5 days from admission to surgery was 156 patients (78 subjects in the experimental and control groups, respectively). This calculation was made using v. 7.10 (June 2010) of the GRANMO calculator (available at: https://www.imim.es/ofertadeserveis/en_granmo.html).

#### 2.9.2 Data analysis

The main analysis of efficacy will be made “per protocol”, but an analysis on an Intention-To Treat (ITT) basis will be done to test the consistency of the results. In the “per protocol” analysis will be included patients that have received the corresponding test for platelet function and have been undergoing surgery. In the ITT, analysis will be included all patients randomized independently if they have received the test or the surgery.

The 2 groups will be compared to ensure that there are no significant differences in the clinical variables of interest. Student's *t* test will be applied to check for between-group differences in the main study endpoint (mean time from admission to surgery). The specific statistical analysis of the baseline characteristics will depend on the nature of the evaluated variables. For categorical variables, we will use the chi-square test with the corresponding contingency table. Ordinal or quantitative variables with clear non-normal distribution will be analyzed using the nonparametric test for more than 2 groups (Kruskall-Wallis), including medians with ranges for each group. The *t* test will be used to assess quantitative variables that meet normality criteria, with mean values and SD provided for each group.

All variables showing statistically significant or clinically relevant differences between the groups on the initial comparison will be entered into a multivariate model to confirm that the differences relative to the study's main endpoint remain significant on an analysis of covariance.

The EQ-5D-5L measures 5 dimensions (mobility, self-care, usual activities, pain/discomfort, and anxiety/depression) related to quality of life. Each dimension has 5 levels of severity (no problems, slight problems, moderate problems, severe problems, and extreme problems) which are scored from 1 (no problems) to 5 (extreme problems) points, with the maximum score of 1 indicating a state of full health. The partial results on each dimension and the overall score will be calculated using the scoring algorithms developed by Ramos-Goñi et al.^[24]^ Because this questionnaire yields ordinal data, nonparametric tests will be used to analyze the comparisons. Between-group differences will be analyzed using the Mann-Whitney and Kruskal-Wallis tests.

The EQ-VAS evaluates patient-perceived health status. This is a vertical scale ranging from 0 to 100. An ANalysis Of VAriance (ANOVA) will be performed.

For the economic analysis, we will calculate the costs in Euros (€) based on the prices of the local health system. For the cost-effectiveness analysis, the costs of the study intervention will be compared to the incremental results, measured in natural units. Cost-effectiveness will be measured by the time from hospital admission to first mobilization after surgery. If the results in the 2 groups are similar, we will perform a cost minimization analysis. Data will be converted into a unit-cost evaluation to determine average cost per patient. We will conduct a comparative economic evaluation of the 2 study alternatives. Confidence intervals will be used to determine the presence of significant differences.

To perform the cost-utility analysis, we will assess Quality-Adjusted Life Years (QALYs)—measured with the EQ-5D-5L—and cost per QALY, calculated incrementally (Δ cost/Δ QALYs) in each group.

We will perform a deterministic sensitivity analysis to evaluate the robustness of the results. The level of significance for all statistical analyses will be set at 5%. The SPSS statistical software program (v. 24) will be used to perform the statistical analyses.

### 2.10 Serious adverse event (SAE)

The organization of the study has established a circuit of notification for serious adverse events. An SAE is defined as any adverse event that:

1. causes the death of the patient;
2. is considered life-threatening;
3. requires hospitalization;
4. prolongs an existing hospitalization;
5. causes permanent or significant disability; or
6. causes an abnormality or congenital malformation.

### 2.11 Data monitoring

The study will be monitored by the Spanish Clinical Research Network (SCReN) which is a public organization independent from the sponsor and researchers to ensure the following: appropriate inclusion of patients, availability of the informed consent form for all patients, protocol adherence of the researchers, and the application of standards of good practice in research in each center. At least 3 visits per center have been planned.

### 2.12 Ethics and dissemination

This trial will be conducted in accordance with the latest revision of the *Declaration of Helsinki* governing standards for good clinical practice.

All patients will be provided with a detailed, easy to understand (i.e., non-technical) written and oral explanation about the nature, scope, and possible consequences of participation in this trial. All patients will be required to provide written informed consent to participate. All patients will receive a document with detailed information about the trial. Patient confidentiality will be guaranteed because the data will be de-identified. This study has been approved by the scientific research ethics committees at all participating hospitals.

There is a policy insurance that cover patients for any adverse event during the study.

The protocol has been presented at national and international conferences. The results of the clinical trial will be published independently and transparently, regardless of the results. The authors of the publication will be the same researchers who have participated in the design and/or execution of the study.

## 3 Discussion

This clinical trial will assess the efficacy and cost-effectiveness of platelet-function monitoring to enable earlier surgery for femoral fracture patients on chronic antiplatelet drugs under spinal anesthesia. This trial will also determine the impact of this treatment strategy on the length of hospital stay and complications.

Femur fractures require urgent surgical treatment, preferably within 48 hours.^[9]^ If the fracture is not treated within this period, research shows that there may be a substantial increase in morbidity and mortality rates, length of hospital stay, and healthcare costs.^[3,5,18–20]^

A range of different antiplatelet agents are available. Inter-individual variability in response to the effects of these drugs is high, particularly for bleeding. For this reason, the risk of hemorrhage should be evaluated on an individual basis. Most patients with a proximal femur fracture are elderly and polypharmacy in these patients is common, potentially causing gastrointestinal absorption disorders, metabolism alterations, and drug interactions. As a consequence of polypharmacy, platelet function may be normal in some patients on antiplatelet therapy due to interactions with other drugs.

After discontinuation of antiplatelet therapy, invasive surgical procedures must be postponed until functional platelet levels have recovered. However, the time required to ensure a sufficient margin of safety depends on the pharmacokinetics of the particular medication. Neuraxial anesthesia is contraindicated in patients on antiplatelet therapy in whom early surgery is indicated but whose platelet function is unknown. The usual approach in these patients is to perform surgery under general anesthesia. However, a recent study involving patients with femoral fracture found that, compared to neuraxial anesthesia, general anesthesia was associated with higher rates of in-hospital mortality and a longer hospital stay, but without significant differences in mortality at 30 days after surgery.^[16]^ Nevertheless, more studies are required to confirm those findings.

Given the risks of general anesthesia in these patients, there is a growing interest in the use of platelet function testing, as evidenced by recent clinical guidelines suggesting that physicians should consider using these tests in selected patients.^[14]^ The value of determining preoperative platelet function is to optimize the timing and safety of early surgery under spinal anesthesia.

Few studies have assessed the effect of early surgery on proximal femur fracture in patients on chronic antiplatelet therapy. Nevertheless, the available evidence shows that patients receiving chronic antiplatelet medications who undergo early surgery present lower postoperative hemoglobin levels (around 1 g/L) than patients in which surgery is delayed, suggesting that patients on antiplatelet therapy experienced a greater amount of blood loss.^[25,26]^ However, the findings of those studies must be interpreted cautiously given the relatively small sample sizes (<50 patients) and thus limited statistical power to identify significant and clinically relevant differences. One randomized clinical trial measured platelet function in patients with and without antiplatelet drugs, but did not use these data to determine whether early surgery was indicated or not.^[27]^

To our knowledge, the present study is the first randomized clinical trial to assess a strategy based on determining platelet function in order to reduce the time from admission to surgery under spinal anesthesia in patients with femur fracture. In this study, we will assess patient quality of life and also conduct cost-effectiveness and cost-utility analyses to determine the impact of this strategy on these variables. In addition, we will compare the PLTwork and PFA-100 systems to assess their level of agreement. The main limitation of this trial is the lack of treatment blinding. A second limitation is that only an exploratory analysis of secondary outcomes (morbidity and mortality) will be performed.

## 4 Conclusions

Determining preoperative platelet function in patients on chronic antiplatelet therapy may shorten the time from emergency department admission to surgery with regional anesthesia. This individualized approach may increase the safety of the surgical procedure by minimizing the risk of bleeding and anesthesia-related complications.

## Acknowledgments

We thank Ms. Andrea Cervera and Mr. Bradley Londres for editing the manuscript. Dr. Angela Merchan is a PhD candidate in Methodology of Biomedical Research and Public Health Program, Universitat Autònoma de Barcelona, Spain.

## Author contributions

Developed the trial design and obtained funding: Martinez-Zapata Maria Jose, Moral Victoria, Rodriguez Mireia, Anaya Rafael, Gil José María, Millán Angélica, Delgado Claudia Erica, Antoijoan Rosa María, Vilalta Noèlia, Reguant Francesca.

Revised it critically and approved final version: All authors.

**Conceptualization:** Moral Victoria.

**Data curation:** Anaya Rafael, Rodriguez Mireia, Gil José María, Millán Angélica, Vilalta Noèlia, Reguant Francesca, Guilabert Patricia, Blanco Domingo, Merchan-Galvis Angela.

**Formal analysis:** Martinez-Zapata Maria Jose.

**Funding acquisition:** Anaya Rafael, Rodriguez Mireia, Gil José María, Vilalta Noèlia, Delgado Claudia Erica, Antonijoan Rosa María, Reguant Francesca, Guilabert Patricia, Martinez-Zapata Maria Jose.

**Investigation:** Anaya Rafael, Rodriguez Mireia, Gil José María, Moral Victoria, Millán Angélica, Vilalta Noèlia, Delgado Claudia Erica, Antonijoan Rosa María, Reguant Francesca, Guilabert Patricia, Blanco Domingo, Merchan-Galvis Angela, Mateo José, Martinez-Zapata Maria Jose.

**Methodology:** Delgado Claudia Erica, Antonijoan Rosa María, Merchan-Galvis Angela, Martinez-Zapata Maria Jose.

**Project administration:** Martinez-Zapata Maria Jose.

**Resources:** Martinez-Zapata Maria Jose.

**Supervision:** Martinez-Zapata Maria Jose.

**Writing – original draft:** Merchan-Galvis Angela, Martinez-Zapata Maria Jose.

**Writing – review & editing:** Anaya Rafael, Rodriguez Mireia, Gil José María, Moral Victoria, Millán Angélica, Vilalta Noèlia, Delgado Claudia Erica, Antonijoan Rosa María, Reguant Francesca, Guilabert Patricia, Blanco Domingo, Merchan-Galvis Angela, Mateo José, Martinez-Zapata Maria Jose.

Martinez-Zapata Maria Jose orcid: 0000-0002-5836-1138.
