# Evaluation of a strategy to shorten the time to surgery in patients on antiplatelet therapy with a proximal femur fracture (AFFEcT Study): Study protocol for a multicenter randomized controlled clinical trial: Erratum

**DOI:** 10.1097/MD.0000000000016316

**Published:** 2019-06-28

In the article, “Evaluation of a strategy to shorten the time to surgery in patients on antiplatelet therapy with a proximal femur fracture (AFFEcT Study): Study protocol for a multicenter randomized controlled clinical trial”^1^, which appeared in Volume 98, Issue 19 of *Medicine*, Dr. Gil José María's affiliation appeared incorrectly and should be Anesthesiology Service, Hospital de la Santa Creu i Sant Pau, IIB Sant Pau, Spain.

Dr. Delgado Claudia Erika's name appeared incorrectly as Claudia Erica.
